# Incorporating Worker Awareness in the Generation of Hazard Proximity Warnings

**DOI:** 10.3390/s20030806

**Published:** 2020-02-02

**Authors:** Kelsey Chan, Joseph Louis, Alex Albert

**Affiliations:** 1 JE Dunn Construction, Portland, OR 97209, USA; kelsey.chan@jedunn.com; 2 School of Civil and Construction Engineering, Oregon State University, Corvallis, OR 97331, USA; 3 Department of Civil, Construction, and Environmental Engineering, North Carolina State University, Raleigh, NC 27607, USA; alex_albert@ncsu.edu

**Keywords:** construction safety, hazard proximity, sensors, worker awareness, automated warnings

## Abstract

Proximity warning systems for construction sites do not consider whether workers are already aware of the hazard prior to issuing warnings. This can generate redundant and distracting alarms that interfere with worker ability to adopt timely and appropriate avoidance measures; and cause alarm fatigue, which instigates workers to habitually disable the system or ignore the alarms; thereby increasing the risk of injury. Thus, this paper integrates the field-of-view of workers as a proxy for hazard awareness to develop an improved hazard proximity warning system for construction sites. The research first developed a rule-based model for the warning generation, which was followed by a virtual experiment to evaluate the integration of worker field-of-view in alarm generation. Based on these findings, an improved hazard proximity warning system incorporating worker field-of-view was developed for field applications that utilizes wearable inertial measurement units and localization sensors. The system’s effectiveness is illustrated through several case studies. This research provides a fresh perspective to the growing adoption of wearable sensors by incorporating the awareness of workers into the generation of hazard alarms. The proposed system is anticipated to reduce unnecessary and distracting alarms which can potentially lead to superior safety performance in construction.

## 1. Introduction

Construction workplaces have been recognized as being one of the most dangerous working environments. For example, the construction industry consistently reports one of the highest number of fatalities across all industries in the United States annually [1]. Much of these injuries resulted from incidents involving falls and contact-with-objects (e.g., struck-by incidents and caught-between incidents) such as heavy equipment [2]. In fact, these incidents have consistently accounted for over 60% of the fatalities reported in the construction industry since 2003 [1]. To address these disproportionately high injury rates, the construction industry has adopted several standard safety management practices (e.g., job hazard analyses, pre-task safety planning). These practices typically rely on the ability of workers to recognize and manage safety hazards [3]. However, recent investigations from around the world have demonstrated that workers often fail to recognize a disproportionate number of safety hazards [4,5]. There is also evidence to suggest that the bulk of these hazards remains unrecognized and unmanaged due to human factors, including cognitive overload, attentional lapses, and distractions [6,7,8].

To address these human-related shortcomings, there has been much interest in leveraging various technologies such as virtual reality and sensors to automate and augment human hazard recognition and avoidance. For example, recent research has focused on developing various hazard proximity warning systems using localization systems [9,10,11,12] that alert workers when they get dangerously close to safety hazards that can cause injury. These efforts have demonstrated that workers are able to adopt appropriate hazard avoidance measures after being notified of their proximity to safety hazards such as mobile equipment [13,14].

While the adoption of these interventions can effectively automate and augment human hazard recognition, there are important risk factors associated with their adoption. For example, previous research has shown that workers can become desensitized to these alarms, a phenomenon known as alarm fatigue, when they are repeatedly activated, particularly when they are unnecessary and redundant [13,15,16]. Such errors that are activated in the absence of unsafe conditions are known as false positive errors (or Type I errors), as opposed to false negative (or Type II) errors. The focus of this paper is to reduce Type I errors for proximity warning systems in construction. Such circumstances can arise (1) when a worker is already aware of the hazard but must work in its vicinity to complete planned tasks or (2) the worker has already initiated avoidance measures after having noticed the hazard.

The negative consequence of alarm fatigue in the construction context is that it may lead to situations where workers may ignore legitimate alarms that are indicative of a hazard that can cause injury [17,18]. In other cases, workers may decide to disable the alarm to reduce repeated distraction and nuisance [19]. Alarm fatigue can also be problematic given that alarms are being explored for a wide range of applications (e.g., back-up alarms, gas-leak alarms, emergency sirens, equipment failure, etc.) in the construction industry context; all competing for the limited attentional resource available to workers. Unnecessary alarms can also lead to distractions that interfere with safe work operations and the adoption of timely hazard avoidance measures [8]. Therefore, limiting alerts and alarms to only cases where workers have not already devoted attention to the hazards can be beneficial. 

To address the discussed concerns that are intrinsic of existing hazard proximity warning systems, the purpose of the presented research was to test the value of incorporating the field-of-view of workers as a proxy for hazard awareness in the generation of hazard proximity warnings. Field-of-view is selected as the proxy for awareness and attention in this research based on previous research that established visual fixations on hazards, which is a function of the gaze direction of workers as a significant predictor of whether hazards are recognized [5,20]. Moreover, a number of previous research efforts have adopted the head orientation of individuals in both construction and non-construction settings to capture the visual attention of the individuals [21,22,23,24,25]. Accordingly, the research focused on testing the utility of incorporating the field-of-view of workers through virtual experimentation towards minimizing the generation of false positive or Type I errors in alarms. Based on promising initial findings, the study was extended to develop a wearable sensor-based real-time hazard proximity system that incorporates the field-of-view of workers for real-world applications. Finally, the effectiveness of the improved system was illustrated through several field-based case studies in the context of fall and equipment-related hazards on construction sites. 

## 2. Literature Review

The following sections provide an overview of technological interventions for injury prevention during construction to provide the necessary foundation and rationale for the current study. Research from previous studies that is used in the methodology of this work to develop a rules-based system for proximity related hazards is also provided. Finally, gaps in the current research are delineated to present the justification of the presented work.

### 2.1. Injury Prevention at the Construction Stage

Although injury prevention efforts are increasingly being adopted in the design and planning stages, most interventions are developed for adoption during construction. These efforts are particularly important given that injury prevention efforts adopted at this phase serves as the last defense against hazard exposure and workplace injuries. Examples of interventions that are widely adopted in the industry at the construction phase include safety inspections, job-hazard analyses, safety training, and the use of safety checklist to facilitate hazard recognition and avoidance [3,26]. Unfortunately, these interventions that rely on the ability of workers have not resulted in desirable levels of performance. For example, past research has demonstrated that workers generally fail to recognize a disproportionate number of safety hazards even when traditional hazard recognition methods are adopted in practice [4,5].

To overcome some of the human-related limitations of existing methods, numerous researchers have proposed sensor-based technologies to automate and augment human hazard recognition and avoidance. For example, Authors in [27,28] proposed the use of wearable inertial measurement units (IMUs) that track worker-movement kinetic data to determine areas where falls are more likely. Likewise, Authors in [29] leveraged unmanned aerial systems (UASs) to identify leading edges which can lead to fall injuries. Other such technologies that have potential to significantly make human workers safer on construction sites include highway work zone intrusion alert technologies [30], and the increasing use of wearable devices for construction workers [31]. 

A portion of these technological interventions focus on the development of hazard proximity alert systems. These systems either localize or track workers with respect to potential safety hazards in the work environment. For example, Authors in [32] used a Bluetooth-based low-energy (BLE) detection technology to identify worker locations with respect to safety hazards in workplaces, Authors in [33] adopted a RFID-based real-time locating system (RTLS) to locate workers and construction equipment with respect to high-risk areas, and Authors in [12] used radio-frequency sensing technology to identify possible collisions between workers and equipment. Several other technologies including laser scanning, ultra-wideband (UWB), global positioning system (GPS), and computer vision have been proposed for similar purposes [34,35,36,37]. While these proximity alert systems offer valuable advantages, there are important challenges to their widespread adoption. Among others, the issue of alarm fatigue that can occur due to unnecessary, redundant, and false alarms is particularly problematic given the already discussed concerns of distraction and becoming desensitized to the alarms [16,17,18]. 

In response to this research need, Authors in [17] has sought to use the heading (or movement direction) of workers and hazard sources to reduce false-positive warnings. Here, when workers and/or hazard sources are moving away from each other, the alarm is not activated despite their proximity, as a hazardous exposure is unlikely in such a circumstance. Another factor that contributes to false positive alarms that has not been considered in previous research is worker awareness of the hazard. Consequently, traditional proximity sensors generate alarms even when workers are already aware of particular hazard sources and may have already initiated hazard avoidance measures. 

Apart from these error sources, the type and accuracy of the localization sensors can affect Type I and Type II errors. For example, any positioning error that can result due to the choice or working of particular sensors could lead to the decision that a worker is safe or unsafe purely based on the distance between workers and the hazard sources. In addition, increased communication time between distributed sensors may lead to Type II errors if the alarm is not generated prior to the occurrence of a hazardous exposure or if the worker is not provided sufficient time to adopt evasive action to prevent hazard exposure.

Since the focus of this research is to incorporate worker awareness to reduce Type I alarms, the integration of multiple sensors to increase the context for decision-making was focused on by obtaining information about both the proximity to hazard and the awareness of worker. Therefore, this literature review informs the choice of sensors used in this research for these two aspects based on the comparative analysis of previous research that is summarized in Table 1.

Based on the applicability of available sensors for localization and awareness tacking as summarized in Table 1, GPS was selected for localization of worker and equipment; and IMU was selected for tracking worker awareness in this research. Apart from the sensing technologies, the choice of communication technology was decided based on providing communication in outdoor settings. For this reason, UWB was selected over competing technologies like Wi-Fi and BLE due to the range achievable with UWB (up to 100 m outdoors) and the lack of need for additional hardware infrastructure such as routers. 

### 2.2. Rules for Proximity-Related Hazards

Since this research focuses on proximity related warning for fall and equipment related hazards, it was necessary to obtain rules regarding the minimum threshold distance between worker and hazard to generate warnings. This subsection briefly describes efforts undertaken to obtain these distances from existing studies and reports on the relevant hazards. The reports provided by the National Institute for Occupational Safety and Health (NIOSH) [39] and the Occupational Safety and Health Administration (OSHA) [2] were reviewed for insights regarding recommended practices when working in the vicinity of fall and equipment hazards. Accident reports filed with NIOSH [39] contained detailed summaries of what occurred during previously recorded incidents, who the incidents affected, and recommendations to prevent future incidents of the same nature. Apart from providing recommended best practices, these reports informed the authors’ choice of case studies as will be discussed in subsequent sections. Additionally, tactical field knowledge relating to best practices was obtained by interacting with superintendents and project managers with an average experience of 10 years to obtain information that is specific to a particular project site.

Based on the report analyses and contractor interactions, a range of 10–15 feet for fall incidents and approximately 20 feet between entities for contact-with-equipment incidents were deemed as safe buffers within which a worker needed to be notified of the hazard. A study [9] that concentrated on creating hazard zones for workers-on-foot around pieces of construction equipment was also consulted. This study determined that a range between seven and 21 feet from a dump truck provided a sufficient safe distance based on typical speeds observed on the jobsite. From these reports and literature review, a range of 15 to 30 feet was used for the rules-based system corresponding to the hazards of focus (i.e., fall and equipment hazards) in the current study. While these distance thresholds have been adopted in the current study, they can be altered in accordance with the application needs in practice.

### 2.3. Research Gaps and Study Objectives 

Since the adoption of standard injury prevention efforts have not yielded desirable levels of performance [4,5], new methods such as hazard proximity warning systems that automate and augment human hazard recognition have been developed [9,10,11,12]. Unfortunately, important weaknesses such as alarm fatigue, false alarms, and related adverse outcomes have impeded their widespread adoption [16,17,18]. One potential solution to this important issue is the consideration of worker awareness in the design and development of these proximity alert systems. More specifically, rather than merely just using the distance from the hazard-source as a criterion for generating these alarms; the incorporation of worker awareness can yield substantial benefits in terms of the effectiveness of such alarms. Accordingly, this paper first focuses on evaluating the utility of integrating the field-of-view of workers as a proxy for hazard awareness using a virtual experiment. Based on the initial findings, an improved real-time hazard proximity warning system that incorporates the field-of-view of workers was developed for field applications. The system’s performance was then tested through several case studies in the context of fall and contact with equipment hazards, as will be described in the following Methods and Case Study sections. 

## 3. Materials and Methods

The primary objective of the methodology is to integrate worker-awareness into the generation of proximity-related warnings hazards caused by fall and equipment-contact hazards. The scope of this research is presented in the next section, followed by the description of the methodology itself. 

Scope of Research Application 

The goal of this developed methodology is to reduce the number of false positive alarms (Type I errors) for proximity-related warning by incorporating worker awareness into its generation. In order to do so, the research considers the larger context of the worker–hazard interaction by determining if the worker is already aware of their proximity to particular hazards, which is the primary factor that is targeted in this research. Due to the use of a virtual model that synthesizes information about the hazard on the site, the application scope of the developed system is limited to such hazards whose existence are a-priori known to the safety manager before the operation starts. Also, the workers will need to be equipped with the integrated sensor package that is developed in this research for both fall and equipment-related hazards. Fall hazards that can be warned against include ledge, holes, and trip hazards that are known to the manager and input into the virtual model before the start of the work. Newly discovered fall hazards would need to be uploaded into the virtual model before workers can be warned of them. For equipment, the developed framework will provide a warning to workers if they are in the vicinity of equipment and they are facing away from it. Equipment will need to be instrumented with a localization system that relays their location to the virtual model. The framework is scalable to include multiple workers and multiple hazards. While the methodology is applicable for construction that occurs both indoors and outdoors, the use of GPS as the localization technology renders the presented hardware implementation of the methodology more suitable for outdoor construction due to the inaccuracy of GPS in indoor environments. The presented system can be modified for indoor use if so required, by utilizing a more appropriate localization system such as Wi-Fi or BLE-based indoor positioning to track the location of the workers 

**Figure 1:**
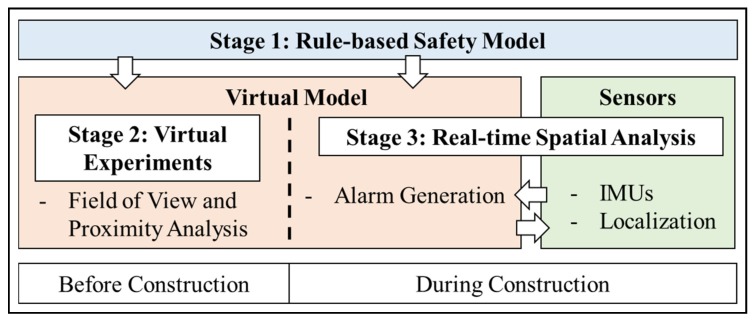
Overview of research methodology for enhanced proximity detection.

The approach taken to incorporate worker awareness in the issuance of proximity warnings in the current study is presented in Figure 1. The following sections describe the three stages in detail.

### 3.1. Stage 1: Development of a Prescriptive Rule-Based Safety Model

A rules-based model that incorporates safe practices for proximity and visibility related hazards was first created to serve as the basis for encoding the field-of-view of workers and generating the safety alarms. To create such a safety model, information on the types of incidents that cause the greatest number of fatalities in the construction industry was gathered from industry safety reports and contractors, as reported in Section 2.2. Regardless of the precise distance used to generate these alarms as finalized by our review of existing literature, the developed methodology in this research allows for the modification of any of these distance parameters as needed for a wider range of potentially hazardous scenarios. Thus, the specific rules that were imposed for the generation of warnings serve to demonstrate the functionality of the system and can be modified based on contractor preferences and experience.

Apart from the proximity related warnings, the worker’s field-of-view was considered in the safety model before a warning was sent out. In order to do this, a warning is sent out only if the worker enters the unsafe distance to a hazard and if the worker has their head turned away from the hazard. In other words, as per the rule-based safety model, an alarm is only generated when the worker’s field-of-view has not focused on the hazard that can cause harm and the worker is within the prescribed unsafe distance. The next section provides a description of the virtual environment where a virtual experiment was conducted to test the utility of incorporating the above discussed rules-based safety model.

### 3.2. Stage 2: Pre-Development Virtual Experimentation

The second stage of the research methodology provides a method to test the efficacy of proposed safety interventions virtually prior to its application in the real-world. Thus, the use of a virtual environment is proposed to test the utility of incorporating the field-of-view of workers in the generation of the alarms on the construction site. This virtual environment provides the researchers with a testbed to visualize and evaluate the interactions between humans and hazards such as mobile equipment without the risk of any injury as would be the case on a real-world site. These virtual experiments were performed using a virtual environment called the Virtual Robotic Experimentation Platform (V-REP) that enabled modeling the interactions between workers and hazards. V-REP is a platform that is intended to virtually model the behavior of robots by creating elements that simulate the behavior of individual sensors, actuators, and control algorithms [40] and has been used to visualize construction operations previously [41]. For this research, V-REP enabled the modeling and visualization of an entire construction site by providing the basic building blocks for the simulation of workers and large construction equipment like trucks and excavators. This modeling ability makes V-REP an ideal testing bed for monitoring complex scenarios that is characteristic of construction operations involving various types of resources working together. The virtual experiment conducted for testing human-equipment interaction is described in the Case Study section.

### 3.3. Stage 3: Enhanced Real-Time Hazard Proximity Warning System

**Figure 2:**
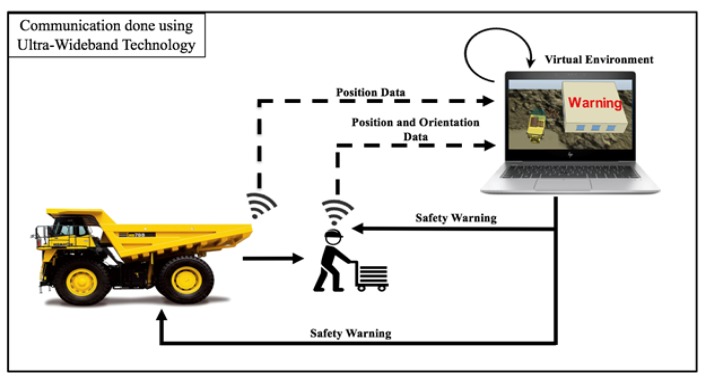
Enhanced Proximity Detection System.

The key features of the improved hazard proximity warning system include (1) the ability to gather location data of both the workers and pieces of equipment in real-time, (2) the capability of projecting or visualizing the location data in real-time within the virtual environment, where the spatial analysis is performed on a continual basis, and (3) the ability to capture and relay worker’s field-of-view into the system. This conceptual system in schematically represented in Figure 2.

The worker’s field-of-view represents the direction of their gaze and contains their visual focus of attention. While this information is typically obtained in controlled settings using computer-vision based eye-tracking systems, the currently available hardware apparatuses have not been approved as meeting the safety standards of the American National Standards Institute (ANSI). Therefore, this research tracks the worker’s field-of-view and gaze direction by using the orientation of the worker’s head, which has been proven to be a suitable substitute for eye-tracking in construction environments as exemplified in by previous studies on tracking user and worker attention [21,22,23,24,25]. This informed our choice of a head-mounted IMU to determine the worker’s field-of-view from their head orientation.

As can be seen, the thee critical components of the enhanced proximity warning system is the hardware that will be necessary to gather the location and orientation data from the entities (i.e., workers and equipment) in the real world, the virtual environment where the spatial analysis is performed to assess proximity and the likely exposure to hazards, and the communication system that is maintained between the hardware in the real-world and the virtual environment for data collection and the issuance of alarms. All communications between the real-world and the virtual environment were implemented to occur via ultra-wideband (UWB) as discussed in more detail below.

#### 3.3.1. Hardware System for Proximity Warning System

**Figure 3:**
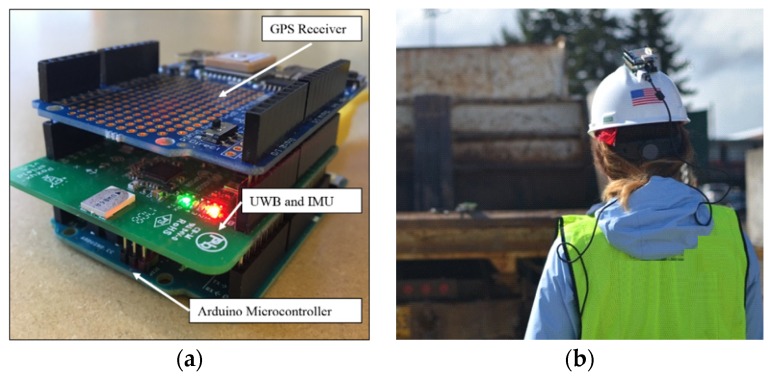
(**a**)Hardware components for streaming data to virtual model. (**b**)Hardware components attached to worker hardhat.

The hardware system attached to the moving entities on the construction site comprises of a GPS receiver for localization, inertial measurement unit (IMU) sensor for orientation, UWB communication module, and an actuator to deliver warnings to workers. All these components were assembled on an Arduino microcontroller as shown in Figure 3a. Figure 3b shows the system attached to a hardhat to be able to track the worker’s location as well as their orientation of gaze. The prototype of the system fits into a three-dimensional bounding box of dimensions 2.7” × 2.1” × 1.9” and is powered by a standalone rechargeable battery.

For field use, apart from the hardware components being attached to the worker’s hardhat, the same sensor hardware package may also be attached to equipment that must be tracked as represented in Figure 2. The information from the sensor package may be transmitted to a UWB receiver that is attached to a computer to update the objects representing the worker and the equipment hazards in the virtual environment. The details of the sensor components and their integration along with the synchronization is provided below.

Hardware Components

The technical details and cost of the GPS module, IMU module, and the UWB communications system are provided here.

1. GPS Module: The GPS module used is the MTK3339 GPS system on a chip which has an update frequency of 10Hz, can track up to 22 satellites on 66 channels, and accuracy of 3m. It operates at a voltage range between 2.8 and 4.3 V. The module was pre-assembled on an Arduino shield that operates under and costs USD 44.95 in the USA.
2. IMU Module: The IMU module used is the Bosch BNO055 Intelligent 9-axis absolute orientation sensor that contains a gyroscope, accelerometer, and geomagnetic sensor, and operates under a voltage range from 1.7 to 3.6V and reports orientation at quaternions and Euler angles. It was embedded in the UWB module described below.
3. UWB Module: The Decawave DW1000, which is a fully integrated single chip UWB transceiver was used to communicate sensor readings from the GPS and the IMU from the real-world to the virtual model and communicate warnings back. It has a communication range of 100m with speeds of 6.8Mbps and operates at 3.3V. The UWB and the IMU modules were integrated in an Arduino compatible shield manufactured by Pozyx and retails for USD 150.

Sensor Integration

The components described in the previous section were obtained as Arduino shields, which are printed circuit boards (PCB) that can be plugged directly on top of compatible Arduino microcontroller boards to extend its functionality. The Arduino Uno board is based on the ATmega328 microcontroller and operates at 5V. Users can upload programs to the board that govern the processing of input and output which is enabled through the provision of general-purpose input/ output pins. Both the GPS module and the UWB module are provided with software libraries that enable the Arduino to directly interface with the capabilities of the respective boards, which greatly simplify the process of integrating multi-modal sensor input. Specifically the *loop()* function for the Arduino board included calls every second to read the latitude and longitude from the GPS module, and the quaternion from the IMU module. These six values (2 from GPS and 4 from quaternion) were appended to a comma separated string, which was then loaded to the UWB module’s TX buffer and then sent to the target UWB module. The receiving module was connected to a laptop workstation through the USB, which enabled serial the communication of the received message from the worker and equipment to the virtual model via serial communication. 

#### 3.3.2. Spatial Analysis in the Virtual Environment

Data streaming from the real-world that is transmitted to the UWB receiver is used to update the positions of the multiple entities in the virtual environment as shown in Figure 2, thereby enabling its scalability. As the location and orientation of the worker or the pieces of equipment changes, the information is updated on a continual basis in the virtual environment. In case, the equipment gets within the unsafe distance as specified by the rules-based model while not being within the worker’s field-of-view, a warning is generated and sent to the worker’ sensor package over the UWB communication system. At this point, the actuator included with the hardware is triggered to provide the worker with a warning.

It is to be noted that the virtual environment will need to be pre-loaded with a model of the site that includes the identified hazards such as any mobile equipment or areas where falls are likely. For hazards such as falls that can occur due to an object on the floor, the hazard can be modeled as a static hazard which does not change position. Accordingly, the position of the hazard will not need to be updated in the virtual environment on a continual basis. However, in the case of dynamic hazards such as mobile construction equipment, the virtual environment will be updated on a continual basis using the data received from the sensors attached to the equipment. 

The developed framework was implemented and tested for various case studies that served to demonstrate its working and test its functionality. The experiments conducted involved the simulation of typical scenarios where this methodology would be applicable. In order to validate the correct working of the system, the system performance was tested by observing both the proximity between the subject and the hazard, and the orientation of the subject’s head relative to the hazard, as well as noting if the alarm was triggered or not based on the rules provided to the system. The experiment was repeated three times for each scenario with the subject instructed to approach the simulated hazard (or have the equipment hazard move towards the subject) while the subject is facing and not facing it. The above method validates the correct working of the methodology and confirms that worker awareness can be considered to prevent the generation of proximity hazards if the worker is aware of them already. 

## 4. Case Study Experiments and Results

Given that the new and improved real-time hazard proximity warning system was developed, virtual and real case studies were conducted to test the working of the system in representative environments. Given the prevalence of falls and contact-with-objects incidents in the construction industry, the case studies focused on simulating these circumstances. First a virtual experiment was conducted to quantify the reduction of false alarms for worker–equipment interaction. For the case studies conducted in the real world, a test subject was equipped with the developed hardware prototype that communicated their real-time location and field-of-view to the virtual environment. This information was in turn used in the virtual environment to perform the spatial analysis and generate relevant alarms whenever appropriate. The case studies are discussed in detail below.

### 4.1. Virtual Simulation of Equipment Interactions

**Figure 4:**
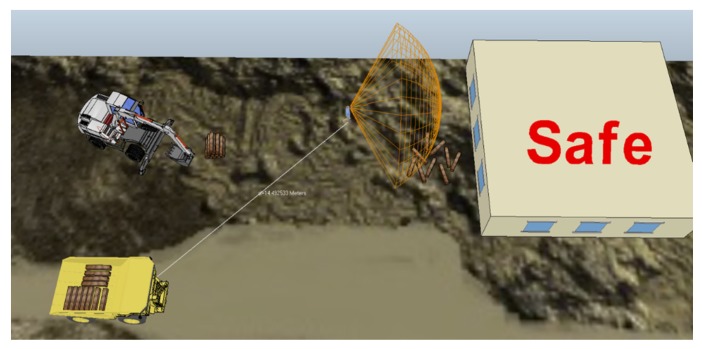
Simulating interactions between workers and equipment using Virtual Robotic Experimentation Platform (VREP).

For the virtual experimentation, the rules-based safety model was implemented within the functionality of the VREP to study and quantify the number of redundant and unnecessary alarms that were issued when the worker’s field-of-view was not considered in the generation of the proximity alarms. A cone-shaped proximity sensor was attached to the head of a virtual mannequin in VREP to simulate the worker’s field-of-view. The simplified geometry of the cone was utilized in this research to represent the typical human’s mid-peripheral field-of-view using a subtended angle of 120 degrees [42]. The worker and other equipment could then be assigned different tasks and paths to move along and a warning is issued based on the rules-based system implemented in VREP. Figure 4 shows a screen shot of two equipment and a worker occupying the same worksite. The orange wireframe shape seen in the figure denotes the worker’s field-of-view. 

For this particular operation that is depicted in Figure 4, the worker was simulated to manually move material from the building to the excavator. Simultaneously, a dump truck carrying additional material navigated around the worksite to unload the material near the building. Such a scenario is representative of the typical level of activity on a worksite wherein humans and equipment work independently but occupy the same workspace. First, an analysis was performed to determine the number of proximity alert warnings that would be issued if only the distance between the entities were used. This was compared with an analysis where both the distance and the worker’s field-of-view was considered. When considering the field-of-view of workers, it was assumed that a warning is unnecessary or redundant if the worker has already fixated on the hazard prior to the onset of the unsafe distance in each of the dump truck cycle. The results of this virtual experiment are summarized in Table 2. As can be observed in the Table 2, when the field-of-view of the worker was considered, the number of redundant alarms reduced by over 77%. The rule-based system was implemented so that the alarm was disabled for a set period (10 seconds in this case) if the worker fixated on the hazard. This reduces the number of alarms generated, which were checked for at a frequency of 1 Hz.

Although the virtual experimentation tested only one scenario, the effort illustrates the following two major points:

1. The virtual platform enables the analysis of operations to capture safety-related information that would be impossible to obtain from real-world experiments. Such an approach offers the benefits of conducting virtual safety-related experiments, without any exposure to real safety hazards that can result in injury.
2. The results of the simulation demonstrate the utility of integrating the field-of-view of workers in reducing the issuance of redundant alarms when the worker has already fixated on or is aware of the hazard. Therefore, the integration of the field-of-view of workers in the generation of these safety alarms can reduce alarm fatigue and unnecessary distractions, which, as discussed earlier, can lead to undesirable outcomes.

Given that the virtual experimentation showcased the utility of incorporating the field-of-view of worker in the issuance of the proximity alarms, the next section extends the use of the virtual environment to provide real-time proximity alerts through the integration of sensors in realistic construction environments

### 4.2. Case Study I: Simulation of Fall Hazard

Falls are a leading cause of both fatal and non-fatal injuries in the construction industry. In fact, estimates suggest that over 350 fatal and 240,000 non-fatal injuries were experienced in construction workplaces in 2017 [43]. Therefore, interventions to reduce the number of falls incidents in the construction industry in necessary. Therefore, the first study that was undertaken simulated the working of the developed hazard proximity warning system in the presence of a fall hazard.

**Figure 5:**
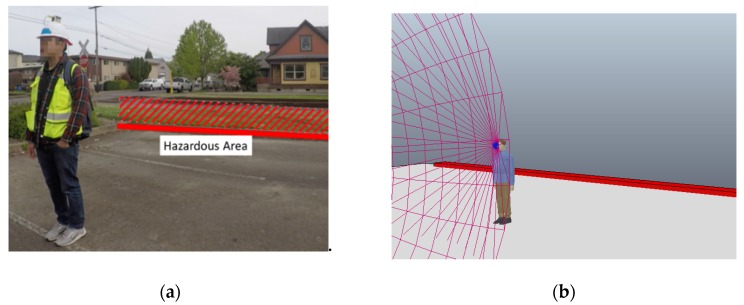
(**a**)Test subject in simulated fall hazard scenario. (**b**)Real-time updated virtual model for fall hazard scenario.

As part of the case study, an open parking lot was designated as the simulated worksite to minimize any risk of actual injury. The curb at the end of the parking lot was considered as the hazardous area as shown in Figure 5a to evaluate the working of the hazard proximity warning system. The curb represents the edge of a top floor in a building under construction. Alternatively, the curb could also be taken to represent an uneven surface which could result in a trip or a fall incident. The simulated work activity was a simple loading and moving task. Incidents in such scenarios are common in the construction industry when workers move in the worksite while paying attention to other work areas or are distracted by external sources (e.g., talking on a cellphone while in the construction workplace). Such circumstances can lead to falls through skylights and even from leading edges [44].

The purpose of the case study was to demonstrate the working of the system where both the worker’s field-of-view or hazard awareness and the proximity to the fall hazard is considered when generating the alarm. For this purpose, the participating worker was instructed to wear the hardhat fitted with the enhanced proximity detection system and perform a series of work tasks that involving him moving about in the work area. As the subject moved about performing the work tasks, the detection system streamed both his location and gaze direction to the virtual model. In Figure 5b, the pink cone represents the worker’s field-of-view and the red cuboid represents the hazardous area. The spatial analysis performed in the virtual model involved checking if the worker was within 4 meters or approximately 13 feet away from the hazardous area and whether the cuboid was not in the worker’s field-of-view. If both conditions were met, then an imminent hazardous situation is detected, and a warning is sent through the UWB communication network back to the worker. The warning was relayed back to the worker in real-time and was conveyed as the flashing of a warning LED on the detection system. The LED was selected purely for demonstration purposes, and a more appropriate actuator could be used to deliver the warning to the workers such as buzzer or an audible signal.

The data streamed to the virtual communication system from the sensors attached to the worker-on-foot and the hazardous area allowed for a precise warning to be sent through the system and back to the worker-on-foot. This warning brought the worker’s attention to the hazard, thereby putting the hazard in the worker’s field-of-view and prevented the worker-on-foot from moving any closer to the hazard. The virtual environment and enhanced proximity detection system was noted to have properly warned the worker and can be used to analyze the unsafe interaction to potentially remove future proximity and visibility related concerns in the context of fall hazards. This fall hazard case study demonstrated the effectiveness of the hardware system and the virtual environment by generating an alarm after considering both the worker’s proximity and awareness of the hazard.

### 4.3. Case Study II: Simulation of Equipment Hazards

Injuries involving contact with objects such as mobile equipment have been responsible for roughly 17% of all fatal and 33% of all non-fatal incidents in the construction industry [45]. Therefore, like the need to focus on fall hazards, interventions targeting contact-with-object incidents are necessary. Accordingly, the usability of the developed hazard proximity warning system was tested in the presence of hazards involving equipment.

In order to simulate worker–equipment interactions that can occur on construction sites, three separate scenarios were examined: (1) worker moving towards a stationary equipment, (2) equipment moving towards a stationary worker, and (3) both worker and equipment are moving along different paths. Each of these separate case demonstrations are first described long with the setup of their respective case studies.

#### 4.3.1. Worker Moving towards Stationary Equipment

There are multiple construction processes that involve situations where a worker may move towards a stationary piece of equipment. For example, a raking or screening job where a worker uses handheld tools to spread and smooth out material laid down by dump trucks or close to pavers is a common activity in construction workplaces. Although a fatal injury may be unlikely in such a scenario, this initial study aided in the verification that the system is capable of detecting the field-of-view of workers and their proximity to other entities on the site. Workers involved in these tasks typically have their heads down, resulting in their field-of-view narrowing and their awareness possibly decreasing when focusing on the task at hand.

**Figure 6:**
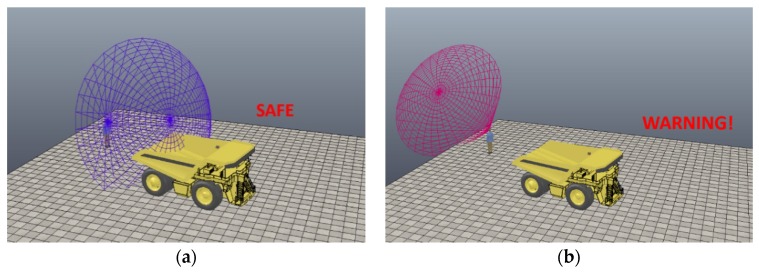
(**a**) Warning not issued because the equipment is now within worker field-of-view. (**b**) Warning is issued when worker is turned away from equipment that is in hazardous proximity.

For this initial study, the parameters for the sensor were set to send a warning when the worker-on-foot was within 30 feet of the truck and when the field-of-view of the worker was not towards the truck. A test subject wearing a hardhat equipped with the enhanced proximity detection system approached a stationary five-ton dump truck. As the test-subjects walked towards the stationary dump truck, the mannequin representing the worker-on-foot in the virtual model also moved in the scene towards the modeled truck. When the test-subject in the real-world approached the truck, and was oriented towards the truck, the worker was deemed as safe and a text projecting “Safe” was visible as shown in the visualization shown in Figure 6a.

However, when the scenario was repeated with the worker and the mannequin not oriented towards the truck (i.e., moving backwards) the cone proximity sensor changed colors and a “Warning” text was displayed in the scene shown in Figure 6b. Given that the alarm was only triggered with the worker was within the set 30 feet threshold and his field-of-view was not towards the truck, the case study demonstrated the reliable functioning of the real-time hazard proximity warning system in reducing redundant and unnecessary alarms.

#### 4.3.2. Equipment Moving towards Stationary Worker

There are multiple scenarios when a worker might be stationary on a construction site and may be unaware of an equipment moving towards them. Such a circumstance can occur when an equipment is operated in the vicinity of other workers. They can occur when a project manager may be distracted by a phone conversation or may have taken a moment to check their handheld device for any updates when in the proximity of heavy equipment. Distracted workers or site personnel are particularly vulnerable to experiencing such a scenario which can potentially translate to a fatal incident [45].

**Figure 7:**
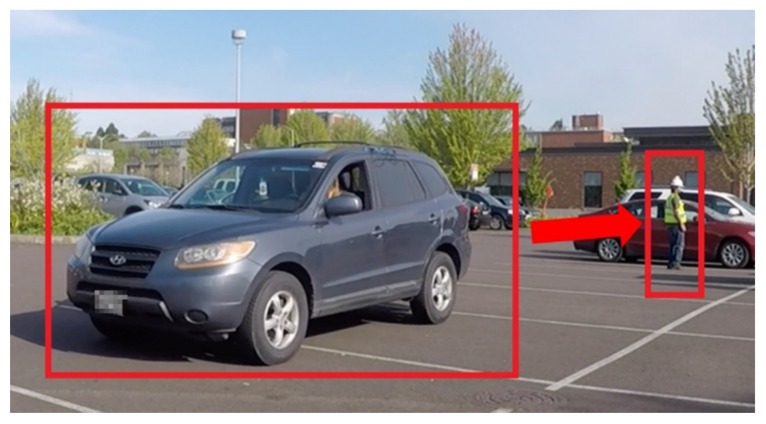
Equipment moving towards worker in simulated worksite.

To evaluate the working of the improved hazard proximity warning system, a similar scenario was simulated. The simulation involved a test subject who was asked to stand stationary in an open parking lot when an equipment, which was a passenger vehicle in this case, approached the test-subject in reverse. It was decided to use the passenger vehicle instead of the five-ton dump truck to ensure the subject’s safety. A range of 30 feet was set as the safe buffer in the virtual scene for the generation of proximity alarm. The experimental setup with the simulated equipment and worker is shown in Figure 7.

The worker stood with their back towards the reversing equipment to represent situations where both entities do not have the ability to see one another on site. Once the vehicle was within the specified hazard distance range and the worker was facing away from the vehicle, the interaction was deemed potentially hazardous and a warning was issued to the worker and the equipment operator. The virtual model corresponding to this scenario is very similar to the previously presented Figure 6a,b. In this case too, the sensor package worked alongside the virtual environment to provide a warning about an impending hazardous interaction on the worksite.

#### 4.3.3. Non-Stationary Worker and Equipment in Vicinity of Each Other

The final scenario for which the system was tested involved a case where the worker and the equipment may be in motion at the same time. Such scenarios are very common in dynamic construction sites where pieces of equipment and workers may work in collaboration or along-side each other. For example, during loading and unloading operations, workers may move behind mobile equipment in preparation for initiating the unloading operation. Alternatively, a mobile equipment may need to back-up to a loading deck which is beside workers involved in a separate construction activity. In such scenarios, workers can be vulnerable to experiencing an incident when they are overly focused on completing a different task which can reduce their situational awareness. While multiple studies (e.g., [15]) have focused on developing proximity detection systems for such scenarios, most experimental efforts have only permitted one of the entities to move during field tests. Therefore, previous testing protocols have often not sufficiently represented the true dynamic nature of construction operations.

**Figure 8:**
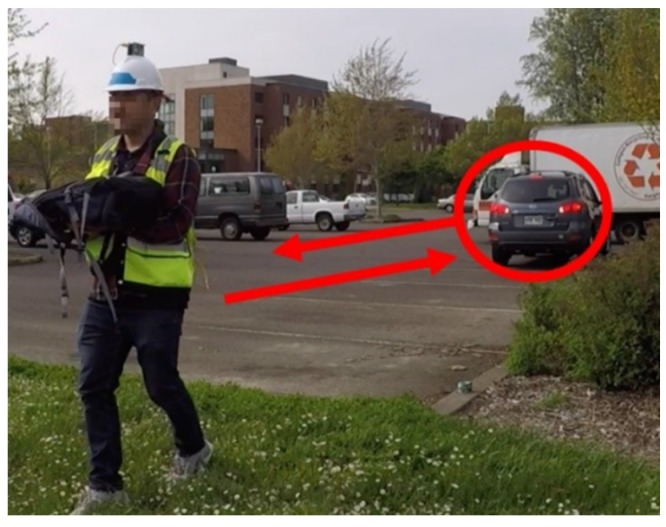
Equipment moving towards non-stationary worker in simulated worksite.

For the purposes of the current effort, an experimental setup similar to the previous two cases was adopted. A typical construction interaction was simulated wherein a test subject was instructed to move back and forth in a designated work area to transport material. While the worker was performing this task, a passenger car that represented an equipment was instructed to back up to the general work area of the worker’s task, as shown in Figure 8.

Both the worker and the equipment were equipped with the sensor package that relayed their information which was projected in the virtual environment. The positions of the virtual worker and equipment were continually updated while the system monitored for instances where the worker was unusually close to the equipment and had their back turned to it. When the equipment was within the specified distance away from the worker, the warning was sent to the worker and the equipment operator to indicate that a potential hazardous situation was imminent.

## 5. Conclusions 

The objective of this paper was to integrate the hazard awareness of workers in the generation of the proximity alarms in order to reduce the occurrence of false positive errors. The contributions, limitations, and suggested future work of the research performed towards this objective are described below. 

### 5.1. Contributions of Research

The study contributes to the body of construction safety literature by demonstrating the utility of integrating the awareness of workers in generating hazard proximity alarms. It is expected that the adoption of the designed system can reduce the number of redundant alarms and promote safe work operations. Specific contributions to the area of construction safety management are described below. 

First, the virtual experiment in the pre-development stages of the study demonstrated that the number of unnecessary and redundant alarms issued by hazard proximity warning systems can be reduced if worker awareness is considered. Such a reduction in the number of redundant alarms can offer several benefits to the construction industry including the reduction of alarm fatigue due to the repeated exposure of workers to redundant alarms, and the reduction of distraction among workers to make them safer and more productive. Likewise, given that alarms are being considered for a number of safety applications (e.g., back-up alarms, gas-leak alarms, emergency sirens, equipment failure, etc.), the reduction of the unnecessary alarms can lead to greater buy-in of the technology from the industry and higher adoption rates.

Second, informed by the findings from the pre-development virtual experiment, a new and improved hazard proximity warning system was developed in the current study that integrates location and the field-of-view of workers using multiple sensors and streamed the information in real-time into a virtual environment. Spatial analysis performed in the virtual environment in real-time indicated when workers were within an unsafe distance to a specific hazard and had not fixated on the hazard, at which time a warning is delivered to the relevant worker. Personalizing warning generation can open the door to the increased use of sensors on construction sites to augment conventional PPE for workers.

Third, the study conducted multiple case studies to evaluate and demonstrate the working of the newly developed hazard proximity warning system. More specifically, the working of the system was demonstrated in the context of both fall and contact-with-objects situations, which included: (1) a worker working alongside a fall hazard, (2) a worker moving towards a stationary equipment, (3) an equipment moving towards a stationary workers, and (4) a dynamic operation involving a moving equipment and worker. In each of the cases, the new hazard proximity warning system functioned as per the design intent, thereby demonstrating the value of the research contributions.

Finally, although the study demonstrated the working of the developed system using a rules-based safety system of limited scope, the system is customizable and expandable to changes in the rules. For example, as already discussed, the minimum distance when the alarm can be triggered can be customized as per the needs of the users or contractors. Likewise, the field-of-view based on which the system suppresses the alarm can be altered to be more narrow or wider (i.e., dimensions of the cone shown in Figure 4) to customize the level of conservativeness needed in generating these alarms. If the dimensions of the field-of-view are made narrower, the system’s conservativeness will improve given that the cone will get closer to only represent areas that the worker directly fixates on.

### 5.2. Limitations of Research

While the presented research makes significant contributions, the following limitations are acknowledged in this paper. The most important limitation is the use of the field-of-view of workers as being representative of hazard awareness. Although past research has demonstrated that the field-of view of workers or the gaze direction is highly correlated with hazard recognition or detection, there may be cases where a worker fixates on a hazard but does not recognize it as a potential safety hazard. Another case that results in a false negative would be if the worker’s view of the hazard was obstructed by another object such as when they are looking at their cellphone. This limitation may be addressed using additional data sources such as brainwaves to determine if workers have processed what they have fixated on as imposing imminent danger to determine if the worker is really aware of the hazard. The current implementation for localization and hazard detection assessments can similarly be supplemented with additional data sources such as the sound from equipment and the reaction of the workers to improve the system performance.

The hardware system was developed as a proof of concept to validate the working of the proposed framework and demonstrate the integration of different sensors and components towards accomplishing the research goal of incorporating worker awareness into the generation of false positive alarms. The system itself is agnostic to the various type of constituent technologies used and these can be changed to optimize the performance of the system. For instance, limitations in UWB include its limited range (~100 m) which may be inadequate for larger construction sites and it also experiences signal attenuation in the presence of metallic objects. To overcome these limitations, future implementations of the system could be used in conjunction with other localization and wireless technologies such as Wi-Fi, Bluetooth Low-Energy (BLE) etc. While this system has adequately demonstrated the accomplishment of the research objective to incorporate worker awareness into the generation of alarms, it still requires further incremental product development in areas such as worker ergonomics and reliability under differing site conditions before it can be deployed in real construction settings. Therefore, the effectiveness and reliability of the hardware system was not evaluated in this paper due to it being outside the scope of this study. 

### 5.3. Recommendations for Future Research

The following recommendations are provided to overcome the limitations discussed above and to further extend this research towards a readily deployable field-ready solution for improving worker safety. Since the case studies presented in this paper were conducted in a relatively controlled, but representative environment, to ensure the safety of the participants while testing a novel integrated sensor system. Future efforts may seek to expand the current efforts to real workplaces after the necessary buy-in and interest is garnered from industry leaders and representatives. Such an effort where there are multiple workers and hazard sources can be used to further validate the usefulness of the currently proposed hazard warning system. It is recommended that future work focus on the statistically evaluating the performance of the system under more realistic conditions after a more robust implementation of the developed system is available. Similarly, multiple virtual simulation experiments must be conducted to provide a more holistic dataset and representation of system behavior in future work prior to utilizing the results from the virtual experiments. 

Additionally, while the current effort used an LED that was attached to the sensor pack to provide warnings to the workers for demonstration purposes, future efforts in the real environment can adopt more suitable actuators to deliver such warnings and well as customize the time duration of warnings and period to ignore false-positive situations. Future research could customize the warning delivered to the worker and examine if the communication of such specifics offers additional advantages. Specifically, research that has been done to determine the real-time activity status of humans and equipment on construction sites (e.g., [46,47]) can be utilized to convey more specific details about the type and severity of the impending hazard.

Finally, future efforts can also focus on linking additional features to the proposed hazard proximity warning system for improving safety. For example, using the concept of the Internet of Things (IoT), future research could further develop the system to trigger the shut-down of an equipment when the proximity between the equipment and worker imposes a significantly higher risk of a collision. In the same manner, future hazard proximity sensing devices can be designed to automatically stop the operation of an equipment when it comes dangerously close to a trench, an open pit, or a restricted work area.

## Figures and Tables

**Table 1:** Summary of Sensor- based Localization and Awareness Tracking for Safety.

**Sensor Type**	**Illustrative Application in Construction**	**Applicability to Current Research Problem**
Global Positioning System (GPS)	Warning generation based on proximity to equipment hazards [11] and to static hazards [13]	GPS localization is appropriate for tracking resources outdoors.
**Sensor type**	**Illustrative Application in Construction**	**Applicability to Current Research Problem**
GPS-aided Inertial Measurement Unit (IMU)	Warning generation based on proximity to equipment, as well as heading and speed of equipment [17]	The use of GPS-aided IMU reduces false positive warning when the equipment is moving away from worker, but this does not consider user awareness.
Bluetooth Low Energy (BLE)-based localization	Proximity warnings for static indoor hazards [32]	Requires the use of static beacons on the site which need to be manually registered. This makes it unsuitable for outdoor and dynamically evolving environments.
Radio Frequency Identification (RFID)-based localization	Real-time resource tracking for construction safety management [33]	RFID localization required multiple readers which need to be manually registered with the site and is therefore not suitable for outdoor worksites that are dynamically changing.
Ultra-wideband (UWB)	Automated tracking of resources indoors for safety monitoring [34,38]	This technique also requires manual registration of anchors for trilateration of tag and is therefore not suitable for outdoor worksites that are dynamically changing.
Vison-based	Warning generation based on proximity to equipment hazards [37]	The presence of occlusions can render this class of techniques unsuitable in dynamic environments.
Inertial Measurement Unit (IMU)	Assessing gait stability [27] and detect near-miss fall [28]	These applications do not use IMUs for localization, but rather to capture motion patterns. IMUs are not suitable for localization due to drift error.
Range-camera awareness tracking	Estimating head orientation of equipment operator to determine their direction of gaze [21]	Range camera and camera can be mounted on equipment, but is unsuitable for tracking worker
Camera-based awareness tracking	Determining attention direction of drivers [23]	
Inertial Measurement Unit (IMU)	Head-mounted IMU was used to determine worker’s visual focus of attention [22]	Appropriate for tracking the awareness and gaze direction of workers on foot.

**Table 2:** Virtual Experimentation Results.

Type of Warning System	Number of Warnings	Percentage
Distance only	71	100%
Distance and worker field-of-view	16	22.54%
Redundant alarms	55	77.46%
